# Chronic Endometritis and Positive Mycoplasma Cultures: Is There a Correlation?

**DOI:** 10.1155/S1064744995000226

**Published:** 1995

**Authors:** Ashwin Chatwani, Paul Nyirjesy, Soheil Amin-Hanjani

**Affiliations:** 1 Department of Obstetrics, Gynecology, and Reproductive Sciences Temple University School of Medicine 7OPD Philadephia, PA 19140 USA; 2 Department of Obstetrics and Gynecology Goddard Medical Associates Brockton MA USA

## Abstract

*Objective:* This study was undertaken to assess the impact of mycoplasma strains (*Mycoplasma hominis* or *Ureaplasma urealyticum*) on the development of chronic endometritis.

*Methods:* Fifty-eight patients with acute pelvic infection were enrolled in this prospective cohort study. Endometrial cultures and biopsies were obtained on admission and 5–7 and 21–28 days after completion of treatment.

*Results:* Of 148 samples, 40 were positive for mycoplasma strains (group A) and 58 were positive for mycoplasma with other pathogens (group B). Twenty-seven samples were positive for other pathogens only (group C). Chronic endometritis was seen in 7 (17.5%), 30 (51.7%), and 10 (37%) in group A, B, and C patients, respectively.

*Conclusions:* The presence of mycoplasma strains in the endometrial cavity was not found to be associated with an increased incidence of chronic endometritis.


Infectious Diseases in Obstetrics and Gynecology 3:3–6 (1995)
© 1995 Wiley-Liss, Inc.

### Chronic Endometritis and Positive Mycoplasma Cultures: Is There a Correlation?

**Ashwin Chatwani, Paul Nyirjesy, and Soheil Amin-Hanjani**

*Department of Obstetrics, Gynecology, and Reproductive Sciences, Temple University School of Medicine, Philadephia, PA (A.C., P.N.), and Department of Obstetrics and Gynecology, Goddard Medical Associates, Brockton, MA (S.A.-H.)*

### Abstract

***Objective:*** **This study was undertaken to assess the impact of mycoplasma strains (*Mycoplasma hominis* or *Ureaplasma urealyticum*) on the development of chronic endometritis.**

***Methods:*** **Fifty-eight patients with acute pelvic infection were enrolled in this prospective cohort study. Endometrial cultures and biopsies were obtained on admission and 5–7 and 21–28 days after completion of treatment.**

***Results:*** **Of 148 samples, 40 were positive for mycoplasma strains (group A) and 58 were positive for mycoplasma with other pathogens (group B). Twenty-seven samples were positive for other pathogens only (group C). Chronic endometritis was seen in 7 (17.5%), 30 (51.7%), and 10 (37%) in group A, B, and C patients, respectively.**

***Conclusions:*** **The presence of mycoplasma strains in the endometrial cavity was not found to be associated with an increased incidence of chronic endometritis.** © 1995 Wiley-Liss, Inc.

Key words

Pelvic inflammatory disease, *Mycoplasma hominis*, *Ureaplasma urealyticum*, endometrial cavity

The precise role of mycoplasma organisms in upper-genital-tract disease in women remains controversial. There is even greater controversy on whether or not mycoplasma in endometrial cultures is associated with endometritis. The report of Horne et al.[1] suggests that this association exists, while Gump and colleagues[2] and Bercovici et al.[3] reported no association between mycoplasma-positive endometrial cultures and endometrial inflammation. The purpose of the present study was to examine prospectively a group of patients presenting with acute pelvic infection and to determine whether the presence of mycoplasma in the uterine cavity was correlated with histologically proven chronic endometritis.

### SUBJECTS AND METHODS

Fifty-eight patients admitted with a clinical diagnosis of acute pelvic inflammatory disease were enrolled in this study. The diagnosis of acute pelvic infection was based on the presence of all of the following: abdominal pain and tenderness, uterine tenderness, cervical motion tenderness, and adnexal tenderness. In addition, 1 or more of the following were present: elevated temperature (>38°C), elevated WBC count (>10,000), elevated sedimentation rate (>20 mm/h), elevated C-reactive protein (>0.0625 mg/dl), or the presence of purulent vaginal discharge. At the time of admission, cervical and endometrial cultures were obtained for *Neisseria gonorrhoeae* and *Chlamydia*

**Address correspondence/reprint requests to Dr. Ashwin Chatwani, Department of OB/GYN, Temple University Hospital, 3401 North Broad Street, 7OPD, Philadelphia, PA 19140.**

Received September 28, 1994
Accepted February 23, 1995

*trachomatis*. Endometrial cultures were also obtained for *Mycoplasma hominis, Ureaplasma urealyticum,* facultative aerobes, and anaerobes. An endometrial biopsy for histologic examination was performed with an endometrial biopsy curette (Pipelle™, Unimar, Inc., Wilton, CT). The endometrial cultures and biopsy were obtained after cleansing the cervix with betadine solution.

Cultures for *N. gonorrhoeae* and *C. trachomatis* were placed on Thayer-Martin plates and Bartel's medium, respectively. For facultative aerobes, cultures were inoculated into BBL port-a-cul medium (BBL, Lockeysville, MD) and then onto sheep blood agar plates, MacConkey agar plates, chocolate agar plates, and V-agar plates. For anaerobes, CDC anaerobe blood agar, CDC anaerobe blood agar with kanamycin and vancomycin, and prereduced PRAS brain heart infusion broth were used. Mycoplasma cultures were placed into Shepard's 10B broth and transferred to the reference laboratory at −70°C on dry ice. The broth was incubated at 37°C under atmospheric conditions. The growth of mycoplasma was suggested by an alkaline shift due to urease activity by *U. urealyticum* or arginine hydrolysis by *M. hominis*. The broth cultures were incubated for 5 days before they were called negative. The positive broth cultures were inoculated on A-8 agar plates, just as the pH indicator began to turn for morphologic identification. The A-8 agar plates were incubated under 5% carbon dioxide. *M. hominis* colonies were identified by their typical "fried egg" appearance and *U. urealyticum* by black colonies.

After successful inpatient treatment with antibiotic therapy (cefoxitin, 2 g IV q 6 h, and doxycycline, 100 mg IV or orally q 12 h until resolution of the symptoms), the patients were discharged on oral medications. Forty-five patients returned for the 1st follow-up visit 5–7 days following the completion of oral treatment and a 2nd follow-up visit 21–28 days after the completion of treatment. The 2nd and 3rd sets of specimens (endometrial cultures and biopsies) were obtained on both occasions.

The endometrial biopsies were examined for evidence of chronic endometritis. The diagnosis of chronic endometritis was based on the presence of any plasma cells. The culture results from each visit were correlated with the histological findings from the biopsy obtained at the corresponding encounter.

Statistical analyses were performed using Epi Info (Centers for Disease Control, Atlanta, GA). The Mantel-Haenszel chi-squared formula was used. When a cell value $<5$ was encountered, a 2-tailed $P$ value was obtained with the Fisher exact test.

### RESULTS

Fifty-eight patients who presented with acute pelvic infection were enrolled in the study. Forty-five of these patients returned for both follow-up visits. Their ages ranged from 17 to 39 years. The mean gravidity and parity were $2.1 \pm 1.7$ and $1.6 \pm 1.1$, respectively. Eighty-one percent were black, 10% were white, and the remaining 9% were Hispanic.

There were 148 biopsies and 148 sets of cultures. At the initial presentation, 7 cultures were positive for mycoplasma only, but only 1 corresponding biopsy showed chronic endometritis. All together, 40 samples were positive for mycoplasma strains alone (23 positive for both *M. hominis* and *U. urealyticum*, 12 positive for *M. hominis* only, and 5 positive for *U. urealyticum* only), but only 7 had evidence of chronic endometritis (5 with both *M. hominis* and *U. urealyticum*, 1 with *M. hominis* only, and 1 with *U. urealyticum* only). Two of 23 biopsies with no growth had evidence of chronic endometritis. The difference in the incidence of chronic endometritis was not significant ($P = 0.33$) between these 2 groups (Table 1). Histologic evidence of chronic endometritis was seen in 30 of the 58 patients with mycoplasma strains and other pathogens and in 10 of the 27 patients with other pathogens alone (Table 1). The incidence of chronic endometritis was not statistically different ($P = 0.20$) in these 2 groups either. The pathogens other than mycoplasma at each visit are presented in Table 2.

### DISCUSSION

In 1966, Hirth et al.[4] demonstrated that mycoplasma was capable of causing salpingo-oophritis in cows. In 1977, Ruben and colleagues[5] demonstrated histologic evidence of severe inflammation of the fetal membranes in cows inoculated with mycoplasma. Gabridge[6] produced histopathology

**TABLE 1. Correlation of mycoplasma cultures with chronic endometritis**

| | No. of biopsies | Chronic endometritis | | *P* |
|---|---|---|---|---|
| | | Present | Absent | |
| Mycoplasma alone | 40 | 7 | 33 | 0.33 |
| No growth | 23 | 2 | 21 | |
| Mycoplasma with other pathogens | 58 | 30 | 28 | 0.20 |
| Other pathogens only | 27 | 10 | 17 | |

**TABLE 2. Pathogens other than mycoplasma**

| Organisms | Admission | First follow-up | Second follow-up |
|---|---|---|---|
| *Neisseria gonorrhoeae* | 34 | 0 | 2 |
| *Chlamydia trachomatis* | 9 | 0 | 1 |
| Non-gonococcal/non-chlamydia | | | |
| Aerobes | | | |
| *Escherichia coli* | 12 | 4 | 0 |
| *Streptococcus* sp. | 10 | 2 | 4 |
| Group D streptococcus | 8 | 4 | 1 |
| Group B streptococcus | 7 | 2 | 1 |
| *Staphylococcus* sp. | 6 | 3 | 4 |
| *Enterobacter* sp. | 3 | 0 | 0 |
| Anaerobes | | | |
| *Bacteroides* sp. | 20 | 14 | 7 |
| *Peptostreptococcus* sp. | 3 | 2 | 1 |
| *Fusobacterium* sp. | 1 | 0 | 0 |

associated with lethal toxicity by inoculating germ-free mice with mycoplasma isolated from humans. Moller et al.[7] were able to demonstrate severe inflammation in the uterine tubes of grivet monkeys inoculated with mycoplasma. These studies suggest a definite role of mycoplasma in infection of the genital tract in animals.

The role of mycoplasma as a genital-tract pathogen in humans is still controversial. Equally unclear is the precise role of mycoplasma as a cause of histologically proven chronic endometritis. Horne et al.[1] first reported subclinical endometrial inflammation in 63% of patients with positive mycoplasma cultures. They reported the characteristic focal accumulation of subacute inflammatory cells beneath the surface epithelium, around a spiral arteriole, or beside a gland in these patients. Bercovici et al.[3] did not find the specific changes described by Horne et al.[1] in 5 endometrial biopsies positive for mycoplasma. Gump and colleagues[2] were not able to demonstrate any signs of inflammation in 10 endometrial biopsies that were positive for mycoplasma. Paavonen et al.[8] did not find a significant association between plasma-cell endometritis and the presence of mycoplasma strains.

In the present study, 7 patients in the initial screening group were positive for mycoplasma strains but only 1 had evidence of chronic endometritis. Overall, there were 40 samples with positive mycoplasma cultures and 23 samples with no growth. The difference in the incidence of chronic endometritis between these 2 groups was not statistically significant. To determine whether mycoplasma had any synergistic effect with other pathogens in causing endometritis, we compared the group of patients with mycoplasma and other pathogens with the group of patients with only other pathogens. The incidence of chronic endometritis was not significantly different between these 2 groups.

One could argue that we should have examined the correlation of mycoplasma cultures with endometritis at each visit instead of grouping the 3 samples together. Since the outcome we were studying was chronic endometritis and not acute pelvic infection, we felt that grouping all similar outcomes together, regardless of when the samples were obtained, was the most appropriate method of examining our data.

The results of the present study demonstrate that the presence of mycoplasma in the uterine cavity is not associated with an increased incidence of chronic endometritis. Furthermore, the presence of mycoplasma and other pathogens in the uterine cavity does not increase the histologic evidence of chronic endometritis. These findings may be due to the limited pathogenicity of mycoplasma or the inability of these organisms to colonize the uterine cavities of some patients.

### REFERENCES

1. Horne HW, Hertig AT, Kundsin RB, Kosasa TS: Subclinical endometrial inflammation and *T-Mycoplasma:* A possible cause of human reproductive failure. Int J Fertil 18:226–231, 1973.
2. Gump DW, Gibson M, Ashikaga T: Lack of association between genital mycoplasmas and infertility. N Engl J Med 310:937–941, 1984.
3. Bercovici B, Haas H, Sacks T, Laufer A: Isolation of mycoplasmas from the genital tract of women with reproductive failure, sterility or vaginitis. Isr J Med Sci 14: 347–352, 1978.
4. Hirth RS, Nielsen RW, Plastridge WN: Bovine salpingo-oophritis produced with semen containing mycoplasma. Pathol Vet 3:616–632, 1966.
5. Ruben Z, Lein DH, Tourtellotte ME: Bovine mycoplasmosis and reproductive failure: An overview of experimental and natural case studies and future outlook. Presented at the 1st International Symposium of Veterinary Diagnosticians, Mexico, January 1977.
6. Gabridge MG: Role of gram negative sepsis in lethal toxicity induced by mycoplasma. J Infect Dis 130:529–533, 1974.
7. Moller BR, Freundt EA, Mardh P: Experimental pelvic inflammatory disease provoked by *Chlamydia trachomatis* and *Mycoplasma hominis* in grivet monkeys. Am J Obstet Gynecol 152:280–286, 1985.
8. Paavonen J, Kiviat N, Brunham RC, Stevens CE, Kuo C, Stamm WE, et al.: Prevalence and manifestations of endometritis among women with cervicitis. Am J Obstet Gynecol 152:280–286, 1985.
